# Best-Evidence Rehabilitation for Chronic Pain Part 2: Pain during and after Cancer Treatment

**DOI:** 10.3390/jcm8070979

**Published:** 2019-07-05

**Authors:** An De Groef, Frauke Penen, Lore Dams, Elien Van der Gucht, Jo Nijs, Mira Meeus

**Affiliations:** 1 Department of Rehabilitation Sciences, KU Leuven, University of Leuven, Herestraat 49, 3000 Leuven, Belgium; 2 Department of Rehabilitation Sciences and Physiotherapy, MOVANT, University of Antwerp, Universiteitsplein 1, 2610 Antwerp, Belgium; 3 Pain in Motion International Research Group, Department of Physiotherapy, Human Physiology and Anatomy (KIMA), Faculty of Physical Education & Physiotherapy, Vrije Universiteit Brussel (VUB), Laarbeeklaan 103, 1090 Brussels, Belgium; 4 Department of Physical Medicine and Physiotherapy, University Hospital Brussels, Laarbeeklaan 101, 1090 Brussels, Belgium; 5 Pain in Motion International Research Group, Department of Rehabilitation Sciences, Faculty of Medicine and Health Sciences, Ghent University, De Pintelaan 185, 9000 Ghent, Belgium

**Keywords:** cancer, pain, rehabilitation, exercise

## Abstract

Pain during, and especially after, cancer remains underestimated and undertreated. Moreover, both patients and health care providers are not aware of potential benefits of rehabilitation strategies for the management of pain during and following cancer treatment. In this paper, we firstly provided a state-of-the-art overview of the best evidence rehabilitation modalities for patients having (persistent) pain during and following cancer treatment, including educational interventions, specific exercise therapies, manual therapies, general exercise therapies and mind-body exercise therapies. Secondly, the findings were summarized from a clinical perspective and discussed from a scientific perspective. In conclusion, best evidence suggests that general exercise therapy has small pain-relieving effects. Supporting evidence for mind-body exercise therapy is available only in breast cancer patients. At this moment, there is a lack of high-quality evidence to support the use of specific exercises and manual therapy at the affected region for pain relief during and after cancer treatment. No clinically relevant results were found in favor of educational interventions restricted to a biomedical approach of pain. To increase available evidence these rehabilitation modalities should be applied according to, and within, a multidisciplinary biopsychosocial pain management approach. Larger, well-designed clinical trials tailored to the origin of pain and with proper evaluation of pain-related functioning and the patient’s pain experience are needed.

## 1. Introduction

Since both prevalence and survival rates of cancer continue to rise, an increasing number of people have to cope with the debilitating effects of this disease and its treatment. Pain is one the most prevalent and persistent problems reported by cancer patients and survivors [1,2]. A recent meta-analysis reported prevalence rates of 55% during cancer treatment and 40% after curative treatment [3]. Pain can interfere with activities of daily life, quality of life and fulfillment of a person’s role in society. Yet, pain during, and especially after, cancer remains underestimated and undertreated [4].

Nowadays, pharmacological treatment is considered the standard approach for treating pain related to cancer (treatment) [5,6]. However, both patients and health care providers are often not aware of other possible rehabilitation strategies and their potential benefits in the management of pain during and after cancer treatment [5,7]. Cancer rehabilitation includes a multidisciplinary and biopsychosocial approach which aims to optimize functioning, well-being and participation of cancer survivors in general, as well as pain relief specifically [7,8]. An important role in the multidisciplinary team is reserved for the physical therapist, at all levels of cancer care (inpatient versus outpatient) and across the whole continuum of complexity of a patient’s pain complaint [8]. Traditional rehabilitation modalities for pain during and following cancer treatment consist of both general (including mind-body exercises) and specific exercises as well as manual techniques to restore physical functioning. Additionally, awareness of the added value of educational interventions in a rehabilitation session has increased substantially and these interventions can no longer be ignored [9,10]. While literature on the beneficial effects of rehabilitation on physical symptoms (such as fatigue, exercise capacity) and general quality of life in cancer patients or survivors is overwhelming, evidence for pain relief in particular is rather scarce in this population. 

In this paper, we firstly provided a state-of-the-art overview of the best evidence rehabilitation modalities for patients having (persistent) pain during and following cancer treatment. Secondly, the findings were summarized from a clinical perspective to facilitate integration from research into daily clinical practice. At last, the state-of-the-art overview was discussed from a scientific perspective. This way, future clinical researchers can build upon this best evidence when designing future trials, implementation studies or new innovative therapies.

## 2. State-of-the-Art

For this paper, we have identified scientific studies using broad search terms including ‘pain’, ‘cancer’ and ‘rehabilitation’ in MEDLINE (PubMed), SCOPUS and Pedro. To minimize selection bias and ensure the selection of high-quality evidence, systematic reviews and meta-analyses were preferred when possible. For the scope of this paper, evidence on rehabilitation modalities for pain during and following cancer was summarized in five categories, being educational interventions, specific exercise therapies, manual therapies, general exercise therapies and mind-body exercise therapies. The focus was limited to cancer patients and survivors with a primary cancer diagnosis and pain during and/or after active cancer treatment. Rehabilitation of advanced and metastatic cancers did not belong to the scope of this paper. Details on the target population, rehabilitation modality, comparator, pain-related outcomes, rehabilitation setting, rehabilitation providers and conclusions regarding the pain-related outcomes can be found in Table 1.

### 2.1. Education

In general, patient education interventions can be defined as “the process by which health professionals and others impart information to patients that will alter their health behaviors or improve their health status” [11].

In the broad field of rehabilitation, the importance of education has increased tremendously over the past years, especially in patients with musculoskeletal pain [12]. In the oncological field, several reviews have summarized the effectiveness of educational interventions on pain intensity, the use of analgesics, side effects and misconceptions on opioids, in patients with pain from active cancer [13,14,15,16,17]. Within the rehabilitation scope of this paper, we found three systematic reviews that had summarized the effectiveness of an educational intervention in the form of individual information, behavioral instructions and advice in relation to management of pain related to cancer (treatment) [16,17,18] (Table 1, Section 1. Education). Comparing educational interventions with usual care, they had found a statistically significant difference in pain intensity in 31% [17], 50% [18] and 52% [16] of the included studies, respectively. Up to 33% [17] and 12% [16] of included studies also showed significant beneficial effects on pain interference with daily activities. Interestingly, Prevost et al. (2016) found that 81% of the studies had significantly improved knowledge and beliefs regarding pain and 45% of studies had improvement in adherence with prescribed analgesics in the education group [16]. In the review of Oldenmenger et al., 68% of the studies showed a significant difference in pain knowledge or barriers, including poor knowledge and misconceptions about pain medication and their side-effects. This last one also evaluated medication adherence and found a statistically significant increase in the education group in 50% of studies [17]. 

However, these studies could neither find a relation between pain knowledge/barriers and pain intensity, nor medication adherence among the included trials reporting both outcomes [17]. Few studies reported effect sizes and despite their significance, these effect sizes were small and of limited clinical relevance in all included studies [18]. Also, response rates are low with only an improvement in pain in 20% of all included patients in the review of Oldenmenger et al [17].

A possible explanation for these rather limited beneficial effects may be the narrow scope of the educational interventions. Indeed, the content of the educational interventions can vary widely among studies and can have different scopes. The emphasis of the educational interventions in these reviews was restricted to a biomedical approach of pain. This is illustrated by the fact that most education was given by (oncology) nurses and medical doctors and mainly covered the consequences of cancer treatment and the pharmacological and medical management of these sequellae. However, considering the increased knowledge of pain pathophysiology, education should additionally incorporate a more biopsychosocial explanation of pain [10], as this has been supported by research in various other chronic musculoskeletal pain populations. This modern educational approach has a broader scope and aims at removing barriers for all aspects of pain management (including self-management and rehabilitation). It targets the patient’s cognitions and knowledge of pain as well as his/her pain-related behavior and thereby aims for a shift from a passive therapy-receiver to an active self-manager [9,10,16,17]. Additionally, education may vary in type (face-to-face, leaflet, video), provider and duration. Furthermore, populations and mechanisms of pain in the included studies were quite heterogeneous, making it unclear whether pain was related to active cancer and/or a consequence of cancer treatment modalities [14,16].

All things concerned, although the effect of educational interventions in a rehabilitation setting seems promising, the ambiguity of its essential components when applied in a cancer population still remains to be further unraveled.

### 2.2. Specific Exercise Therapy

Specific exercise therapy typically includes active and/or active-assisted strengthening, mobilizing and stretching exercises to restore function of the affected region [19]. The literature on the effectiveness of specific exercise for pain in cancer survivors is scarce [20]. A tremendous amount of research has been done on the effect of specific exercises for other upper limb dysfunctions during and after head, neck and breast cancer. Range of motion, upper limb strength and upper limb function in general may be affected after surgery and radiotherapy due to formation of fibrosis and scar tissue, nerve damage, muscle tightness, lymphedema (including axillary cording) and pain [20,21,22]. Specific exercises are indeed prescribed to optimize and/or restore joint and muscle function of the affected region. Reduction in pain is often presumed to occur subsequently. However, pain might be a primary indication for specific exercises as well. In particular, for nociceptive and neuropathic pain at the affected region, specific exercises may aid in increased blood flow, as well as a reduced hyperesthesia, inflammation, biomechanical deficits and muscle spasms [5,23,24]. To our knowledge, only four systematic reviews have summarized randomized controlled trials on the effectiveness of specific exercises in an oncological population that included pain outcome measures [19,20,21,25] (Table 1, Section 2. Specific exercise therapy).

First, in breast cancer patients, three reviews have summarized the effectiveness of different exercise programs compared to usual care or no exercises [19,20,25]. The exercise programs varied in content (mobilization, stretching, strengthening and stabilization exercises) and in duration (timing, frequency and intensity). For (shoulder) pain, with the exception of one study, no differences between two groups were found. This study compared active exercises with a leaflet and showed beneficial effects on pain intensity on a visual analogue scale (0–10) three months (−2.7 95% CI (−3.6 to −1.9)) and six months (−2.5 95% CI (−3.5 to −1.6)) following surgery [26]. Remarkably, almost all studies reported beneficial effects of exercises on shoulder range of motion and/or shoulder function in general.

Additionally, several studies showed no difference in an early or delayed start of specific exercises after surgery for pain incidence and pain intensity up to two years follow-up [19,20]. Comparing a supervised versus non-supervised program [27], differences in neither pain incidence nor intensity were found [19]. 

Another Cochrane review and meta-analysis (including two studies) in patients treated for head and neck cancer pain showed significant beneficial effects of a progressive strengthening training program on pain [21]. However, results were not clinically relevant. 

In conclusion, there is currently no evidence available that supports the use of specific exercise therapy for relieving pain in cancer patients or cancer survivors. Several reasons for this can be postulated. First, these exercise programs were designed to increase physical impairments, including impaired range of motion and strength, and pain was only considered as a secondary outcome. The latter implies that the available trials were not designed to examine the potential for specific exercises on pain relief (i.e., the trials might have been underpowered to detect clinically important changes in pain; the trials included all cancer patients or cancer survivors, while not all patients suffer from clinically relevant levels of pain, in turn decreasing the ability of a treatment to generate important changes). Moreover, most studies only evaluated pain intensity. Other dimensions of pain, or rather pain-related disability, are outcomes of higher clinical relevance and may reflect true effects of specific exercise interventions. Lastly, the underlying mechanism of the patients’ pain complaint was not taken into account when providing the exercise therapy to the patients suffering from cancer or post-cancer pain. Pain during and after cancer treatment can have many origins with different associated indications for exercise therapy. Therefore, prescription guidelines on specific exercises for pain after cancer treatment are not available and it remains to be established which type(s) of exercise therapy (strengthening, mobilizing and stretching exercises) is indicated depending on the predominant pain mechanism at different time points throughout cancer treatment and thereafter. 

The most important message from the limited amount of research is that specific exercises are safe. However, evidence on the best type of exercise, the exact modalities and timing is inconclusive [19,20,21,25].

### 2.3. Manual Therapy

Within the cancer field, studies of manual therapy address passive joint mobilizations and massage therapy. First, manual passive mobilizations primarily aim at restoring joint range of motion trough alleviating capsular restrictions, distracting (soft) tissues and providing movement and lubrication for normal articular cartilage. Additionally, pain relief may be achieved through the activation of mechanoreceptors and stimulation of fast-conducting fibers [28]. The review of De Groef et al. included one RCT on the effectiveness of passive mobilizations after breast cancer surgery [20] (Table 1, Section 3. Manual Therapy). This study indicated beneficial effects of passive mobilizations during the first week post-surgery on long-term prevalence of locoregional pain. However, this one study shows high risk of bias, so results are inconclusive [29].

Second, research on massage therapy in cancer population on the other hand is overwhelming. Massage can be defined as the manipulation of the soft tissues of the body, performed by the hands, for the purpose of producing effects on the vascular, muscular, and nervous systems [30]. Two most recent systematic reviews of RCTs are discussed here (Table 1, Section 3. Manual Therapy). A Cochrane review on massage, including 19 studies of which 5 reported the effects on pain, showed beneficial effects of massage therapy in only one study [31]. Another review found beneficial effects of massage in 79% of the included studies [32]. However, beneficial effects on pain intensity were very small and of limited clinical relevance as illustrated by the meta-analyses: Standardized Mean Difference (SMD) of −0.20 (95% CI, −0.99 to 0.59) for massage versus no treatment and SMD of −0.55 (95% CI, −1.23 to 0.14) for massage versus an active comparator (including attention, usual care, standard treatment, a reading group comparator, and caring presence) [32]. The review indeed concluded in the evidence synthesis that only weak recommendations can be made for massage therapy and that effects are clinically irrelevant [32]. As suggested by the definition of massage therapy, other effects aimed at with massage therapy may be a reduction in anxiety and stress and an enhancement of personal sense of well-being through the effect on body and mind [32]. However, the studies in both reviews were of very low quality and included a mix of primary, advanced, and metastatic cancers; concluding that there is a lack of clear evidence to support the use of massage for pain relief in people with cancer at this moment [31,32]. 

### 2.4. General Exercise Therapy

Exercise can be defined as “any physical activity causing an increase in energy expenditure, and involving a planned or structured movement of the body performed in a systematic manner in terms of frequency, intensity, and duration and is designed to maintain or enhance health-related outcomes” (American College of Sports Medicine). Typically, aerobic and resistance training are considered when discussing general exercise therapy [33,34]. A systematic review of systematic reviews by Stout et al. summarized results of 53 reviews on exercise in cancer populations [35]. They concluded that exercise was beneficial before, during, and after cancer treatment, across all cancer types, and for a wide range of physical outcome parameters. Moreover, exercise was found to be safe during all cancer stages. Several reviews in different cancer populations indeed confirmed the beneficial effect of exercise on quality of life [36,37,38]. Despite this large amount of studies and clear guidelines, no recommendations for using general exercise therapy for the treatment of pain in cancer populations were extracted. 

Frist, Nakano et al. published the most recent meta-analysis limited to RCTs using the European Organization for Research and Treatment of Cancer Quality of Life Questionnaire-C30 (EORTC-QLQ-C30) [39] (Table 1, Section 4. General exercise therapy). They summarized the effect of aerobic and/or general resistance exercises on physical symptoms, including pain, for cancer patients and survivors in any setting. The meta-analysis showed that pain in the intervention group (receiving aerobic and/or resistance exercises) was significantly lower compared to no intervention. However, the effect size was only small (SMD of −0.17 (95%CI, −0.32 to 0.03)) and no differences among the 3 types of exercises interventions could be extracted [39]. Second, two Cochrane reviews of Mishra et al. summarized the effectiveness of exercise interventions on health-related quality of life, including pain, during active cancer treatment [34] and in cancer survivors [33], respectively (Table 1, Section 4. General exercise therapy). During active cancer treatment, no significant effects for pain relief in favor of general exercises were described. In cancer survivors, pooled data of four studies showed beneficial effects of exercise for pain with a small effect size (SMD of −0.29 (95% CI, −0. 55 to −0.04)). Another noteworthy Cochrane review summarized the beneficial effects of general physical activity, including activities as part of occupation, active transportation, household and gardening chores, and recreational activities [40]. Exercise was considered as a subcategory of physical activity. This review showed beneficial effects for a wide range of health-related outcome parameters. For pain however, 9 out of the 63 included trials that reported a pain outcome measurement showed no beneficial effects [40]. 

These reviews summarized the effectiveness of aerobic and/or resistance exercise therapy as an intervention [33,34,39,40]. The type of exercise therapy and specific modality most efficient for pain relief was not clear. In the general population, exercise is considered very important in pain management because of its possible beneficial effect on central pain (inhibitory) mechanisms, the autonomic nervous system, the immune system (anti-inflammatory effect) and subsequent hypoalgesic effect [41,42,43]. However, the response to exercise is more variable in chronic musculoskeletal pain populations and may even result in hyperalgesia [41,42,43]. For cancer populations, even less is known about pain processing during and after exercise therapy, and a possible impaired analgesic response to exercise (therapy) and physical activity. Remarkably, in particular for hormone therapy related arthralgia, which is experienced by up to 50% breast cancer survivors treated with aromatase inhibitors, general exercise therapy holds high value [44,45,46]. Findings from a high-quality randomized controlled trial indicated that 150 minutes per week of aerobic exercise and supervised strength training twice per week can lead to clinically relevant improvements in pain [46].

In conclusion, general exercise therapy is safe and well tolerated, both during and after cancer treatment. However, only limited evidence is available on the beneficial effects for pain relief during and after cancer treatment in general. The exact exercise modalities to ensure this pain relief are not described [33,34,39]. However, a combination of aerobic training and strengthening exercises is recommended for pain relief in patients with hormone therapy related arthralgia [46].

### 2.5. Mind-Body Exercise Therapy

Mind-body exercises intend to enhance the mind’s capacity to positively affect bodily functions and symptoms, including pain, by combining exercises with mental focus [47]. They have gained interest in many fields of rehabilitation, including cancer.

First, pilates has found its way into many rehabilitation practices. One review showed that pilates was statistically more effective than the interventions in the control groups for reducing pain among women with breast cancer, showing a moderate effect (SMD of −0.48 (95%CI, −0.88 to −0.07)) [48] (Table 1, Section 5. Mind-body therapy). However, only women with breast cancer were included. Yoga is becoming very popular in cancer rehabilitation as well, as reflected by the 29 RCTs summarized in the review of Danhauer et al. [49] (Table 1, Section 5. Mind-body therapy). They reported improvements in general quality of life, fatigue, and perceived stress. Pain was investigated only in a very small number of studies showing inconclusive results [49]. Another popular type of mind-body exercises is Tai Chi Chuan. However, for pain in breast cancer survivors, pooled results of three RCTs could not demonstrate beneficial effects (SMD of 0.11 (95%CI, −0.41 to 0.18)) [50] (Table 1, Section 5. Mind-body therapy).

The positive effect of mind-body therapies, in particular yoga in e.g., breast cancer patients, seems more obvious for psychological wellbeing, including stress, anxiety and depression [47,49]. Similarly, for massage therapy, through a reduction in anxiety and stress and enhancement of personal sense of well-being a relief in physical symptoms, including pain, may be achieved [32,47].

Mind-body exercise therapy are often considered as complementary therapeutic interventions and may play an important role in cancer rehabilitation. While pilates seems to have clinically important pain-relieving effects in women with breast cancer, evidence for yoga as a pain-relieving intervention in cancer populations is inconclusive.

## 3. Promising Directions for Clinical Practice

Both clinicians and researchers highlight and emphasize the tremendous need of a systematic follow-up of side-effects related to cancer and its treatment(s), including pain, in order to improve quality of life of cancer survivors. Indeed, besides treatment of cancer itself and follow-up of relapses, a systematic prospective care pathway is minimally required for each cancer patient [51,52,53,54,55]. The key elements of such a care model are (1) a proactive approach to regularly examine and question patients on pain and pain-related disability; (2) providing ongoing assessment during all stages of cancer treatment (often in absence of problems) and (3) uniform efforts to enable early interventions for pain management, including mono- and complex multidisciplinary interventions, both in inpatient and outpatient settings [51,52,53,54,55]. 

A first step in improving clinical practice through clinical care pathways would be early detection and proper diagnosis of pain in cancer. A clear diagnosis of a patient’s pain complaint is a critical step in clinical decision-making. Over the past decades, knowledge on the origin of pain during and after cancer treatment has increased [5,7]. From a tumor-related and a treatment-related classification of pain, there was a major shift to a mechanism-based classification of cancer pain [56,57]. During adjuvant treatment of a primary cancer, the tumour is removed, so it is expected that pain is no more related to cancer, per se. In the early stage of cancer treatment, nociceptive and/or neuropathic pain caused by surgery, radiotherapy and/or chemotherapy is present in most cases [24,58]. At this stage, pain is related to tissue damage and if adequately managed, can be considered as a short-term side effect. In a later stage, when these local effects of the different cancer treatment modalities should have been healed, the initial causes of pain may be overshadowed by sensitization of the central nervous system in a subgroup of cancer survivors [58,59,60]. In this case, pain is no longer related to tissue damage and can be explained by enhanced processing of sensory input (sensitization) within the peripheral and/or central nervous system and by altered pain modulation, leading to so called central sensitization pain or nociplastic pain [61]. Specifically for the cancer population, it is important to recognize that local tissue damage or peripheral mechanisms can continue to contribute to their pain complaint for a long time after completion of acute treatment, together with other sustaining psychosocial factors e.g., postmastectomy pain syndrome and chemotherapy-induced peripheral neuropathy [5] or related to ongoing treatment modalities for several years such as arthralgia related to hormonal therapy [62]. For effective pain management, identification of the predominant pain mechanism is warranted. Clinical guidelines for the identification of the predominant pain mechanism in cancer survivors are available, however not validated [58].

Secondly, through the clinical care pathway, referral for adequate pain management is facilitated [51]. As summarized above, a tremendous amount of research is available on rehabilitation modalities for a wide range of physical symptoms during and after cancer treatment. Despite its high prevalence rates and high disabling impact, in many studies “pain” was not used as outcome measure. Too often pain is considered as “being part of” cancer survivorship, resulting in minimal effort to detect pain and referral for adequate treatment. Although research is limited, the reviews described above point towards promising pain-relieving effects of rehabilitation modalities for pain management during and after cancer treatment. However, effect sizes are only small to moderate and research is limited to mostly breast cancer populations. Based on this, the following modalities can be carefully recommended in general. Firstly, both at the start and during a rehabilitation program, the added value of an educational intervention based on modern pain (neuro)science—including a biopsychosocial explanation of pain—should be considered to remove barriers for rehabilitation and promote adequate pain behavior and cognitions [10]. In particular, in patients with maladaptive pain beliefs and behavior, an educational intervention is warranted to explain pain and how different therapy modalities can potentially influence this. Correct interpretation of symptoms during treatment will facilitate shared decision-making and further therapy adherence. Secondly, currently no evidence supports the pain-relieving effect of specific exercises and mobilizations during and after cancer treatment. Whether these rehabilitation modalities have a role in particular for acute nociceptive and neuropathic pain related to joint and muscle dysfunctions at the affected region should be further investigated [20,21,22,24]. Thirdly, general exercise therapy may result in pain relief. However, more research is needed on the modalities (type, frequency, intensity and duration) to increase effect sizes and ensure reduction in pain and/or pain-related disability and avoid pain flares after exercise [33,34,39]. Indeed, in a subgroup of cancer survivors with predominant nociplastic pain, endogenous pain modulation may be impaired and alter the response to both specific and general exercise therapy. However, it is important to note and explain that rehabilitation interventions, including exercise therapy, are still safe both during and after cancer treatment. In particular, for cancer survivors with hormone therapy related arthralgia, general exercise therapy is recommended [46]. At last, mind-body interventions, including general exercises such as yoga and pilates, might have a complementary role. However, besides the pain-relieving effects of pilates in women with breast cancer, study results are inconclusive. It has been argued that the pain-relieving effect occurs through a reduction in anxiety and stress and enhancement of personal sense of well-being [32]. The evidence on the influence of various psychosocial and emotional factors on the (persistence of) pain has increased past decades [9,63]. Especially in cancer populations, the cancer diagnosis, treatment but also the fear of cancer reoccurrence can induce stress, depression and anxiety among others [64]. As proposed for the educational interventions, a biopsychosocial explanation of pain is necessary. Therefore, mind-body therapies fit within this approach and may be valuable modalities to address pain and its psychosocial sustaining factors. The remark has to be made to what extent these mind-body therapies belong to the rehabilitation domain. Other interventions, e.g., mindfulness, mediation, acupuncture, … are often considered as complementary mind-body interventions as well. However, since the element of bodily movement in these interventions is not apparent, they are beyond the scope of this paper. Given the multiple cancer treatment modalities, pain is often not the only side effect during and after cancer treatment. When initiating rehabilitation modalities for pain, in particular exercise therapy, other comorbidities should be taken into account when developing a treatment plan. Fatigue is often associated with pain and vice versa, and may hamper regular performance of general exercise. Several cancer treatments have a toxic effect on the cardiovascular system among others, leading to decreased exercise tolerance. When establishing an exercise program, this has to be taken into account [65]. 

Pain management during and after cancer treatment is not restricted to pharmacological therapy and rehabilitation interventions. Other disciplines should be part of the rehabilitation team and multidisciplinary treatment should be provided if necessary. Increased stress, anxiety and sleep disturbances have been described to interfere with pain and/or pain-related disability and therefore should be addressed if necessary [9,66]. Social workers may be important to address problems with participation in society [67]. Other lifestyle interventions, including nutrition, smoking and excess alcohol consumption, have been proposed to improve cancer survivorship and quality of life and therefore their role in pain relief should be considered as well [68]. 

Additionally, pioneering studies are emerging on the use of eHealth in the cancer population. Applications for symptom monitoring, including cancer pain, are already available and show promising results [17,69]. These applications may also increase the accessibility to educational resources and self-management strategies [69]. A recent review confirmed that applications supporting self-management improve pain and fatigue outcomes in cancer survivors [70]. In line with this, telecoaching interventions may be of value to increase adherence to specific exercise programs [71] and/or physical activity in general in cancer patients [72]. These technological highlights keep manifesting, but researchers warn that high-quality studies are currently still ongoing and these interventions should be developed and tested properly before being recommended [69].

## 4. Promising Directions for Research

Current state-of-the art rehabilitation for pain during and after cancer treatment is limited. When positive pain-relieving effects for rehabilitation interventions are found, effect sizes are most often small to moderate. Different explanations can be given for this. In clinical practice different interventions are combined, which is an important strength of rehabilitation for pain, but unfortunately hard to translate into research. Additionally, pain relief from a comparative intervention, standard intervention or even no intervention may occur and result in small effect sizes as well. Typically, responders and non-responders can be identified in clinical trials, especially when the intervention is not tailored to e.g., the predominant pain mechanism. This may result in overall small effect sizes at group level. Therefore, a balance between a pragmatic approach and highly standardized conditions is needed in research. In the following paragraph, items that should be considered in further research are discussed. 

Firstly, both from a clinical and scientific perspective, it is highly important to correctly diagnose pain and identify the predominant pain mechanism of a patient’s pain complaint. Clinical guidelines for this purpose are described, however not validated [58,73]. Several studies did not clearly describe whether pain was related to cancer itself or whether it was a side effect of the different treatment modalities. Pain can be a symptom of cancer. However, pain due to cancer often means it has already metastasized. For this paper, the focus was limited to cancer patients and survivors with a primary cancer diagnosis and pain during and/or after active cancer treatment, so it is expected that pain is no more related to the cancer itself. In future studies, this should be specified when diagnosing pain in cancer patients and survivors. Associated with this, due to the prolonged side effects of certain treatment modalities, e.g., radiotherapy and hormone therapy, it is in many cases difficult to distinguish whether a patient’s pain complaint is still related to local tissue damage (nociceptive and/or neuropathic pain) or rather to altered pain processing in nociplastic pain without dominant peripheral input. Studies on the effectiveness of rehabilitation modalities tailored to the predominant pain mechanism might result in larger effect sizes [58,74].

Secondly, besides a proper diagnosis of a patient’s pain complaint in order to tailor rehabilitation modalities, a comprehensive pain assessment is warranted. The Initiative on Methods, Measurement, and Pain Assessment in Clinical Trials (IMMPACT) recommends six core outcome domains that should be considered when designing clinical trials on pain management. These domains include: (1) different dimensions of pain; (2) physical functioning; (3) emotional functioning; (4) participant ratings of improvement and satisfaction with treatment; (5) symptoms and adverse events; and (6) participant disposition [75]. Indeed, in order to unravel the concept of pain, outcomes should not be limited to the impairment itself, but also include pain-related functioning. Moreover, it is argued that rehabilitation modalities and other interventions should focus on improvement of daily functioning and pain-related disability, which will ultimately lead to reductions in patient’s pain intensity (or at least the debilitating nature of pain). On the other hand, generic outcome measures such as general quality of life may lack responsiveness to detect (more subtle) changes in pain [16,75]. Additionally, various psychosocial factors play an essential part in the pain experience and the degree to which someone perceives their pain as disabling. The effect of rehabilitation interventions on psychosocial outcomes and possible moderating and mediating role of these factors in response to treatment for pain during and after cancer treatment should be explored in future studies. At last, to explore the duration of response and sustainability of rehabilitation interventions for pain relief, an adequate follow-up period should be provided.

Thirdly, besides the high burden for the patient in the first place, the socio-economic impact of pain during and after cancer treatment should be investigated. The number of people with long-term sick leave or reduced working hours after cancer continues to rise. Pain, low (perceived) physical functioning and low self-efficacy are factors associated with delayed return to work [76,77]. Additionally, rehabilitation entails a substantial financial cost for the health care system. Increasing the effectiveness of rehabilitation for pain may decrease the number of rehabilitation and costs associated with other more expensive pain management strategies. Therefore, proper health- and socio-economic analyses are needed to change practice. 

Fourthly, technological developments in rehabilitation should not be ignored, as they may lead to innovative treatment avenues for cancer survivors as well. For example, preliminary study results show that effects of virtual and augmented reality for rehabilitation of phantom limb pain [78], (neuropathic) pain related to multiple sclerosis [79] and spinal cord injury [80] and pain in children [81] are promising. In cancer patients, it may be valuable to distract patients from pain, to increase motivation and participation with a better response to treatment as a result. This may be an interesting research topic in the future.

At last, an overwhelming amount of evidence is available in certain cancer populations, e.g., breast cancer. More trials in other cancer populations, such as colon and gynecological cancers are needed before any general recommendations can be given. Additionally, rehabilitation for pain in advanced cancers, palliative settings and populations with social disparities may warrant a different approach and thus needs further investigation. 

## 5. Conclusions

While literature on the beneficial effects of rehabilitation modalities for symptoms such as fatigue, exercise tolerance and general quality of life is overwhelming, evidence for pain relief during and after cancer treatment is rather scarce. In conclusion, best evidence suggests that general exercise therapy has small pain-relieving effects. Evidence for mind-body exercise therapy in breast cancer is promising given the moderate effect size. At this moment, there is a lack of high-quality evidence to support the use of specific exercises and manual therapy at the affected region for pain relief during and after cancer treatment. No clinically relevant results were found for educational interventions restricted to a biomedical approach of pain. To increase available evidence, these rehabilitation modalities should be applied according to and within a multidisciplinary biopsychosocial pain management approach. Larger, well-designed clinical trials tailored to the origin of pain and with proper evaluation including pain-related functioning and other outcomes related the patient’s pain experience are needed.

## 6. Clinical Implications

- Rehabilitation modalities, including manual therapy, specific and general exercise therapy, are safe and well tolerated during and after cancer.
- Evidence for pain relief is scarce but promising.
- Despite the unclarity of essential components of education to improve pain, its role in rehabilitation during and after cancer may be crucial for pain relief.
- Mind-body interventions including e.g., pilates may be complementary.

## 7. Research Agenda

- Distinct prescription guidelines for specific and general exercise therapy according to the FITT principles should be explored.
- Validated guidelines for the accurate identification of the predominant pain mechanism in cancer are warranted.
- The effectiveness of rehabilitation tailored to the predominant pain mechanism should be investigated.

## Figures and Tables

**Table 1:** Detailed best evidence table.

Author, Year (Design)	Target Population	Rehabilitation Modality	Comparator	Pain-Related Outcomes	Rehabilitation Setting	Rehabilitation Providers	Conclusion
**1. Education**							
Oldenmenger et al. 2018 (Systematic review of RCTs)	- adults - solid malignancies - cancer-related pain	Educational intervention: information, behavioural instructions + advice (by verbal, written, audio- or videotaped or computer-aided modalities)	Usual care or active control intervention	- pain intensity (NRS or VAS) - pain interference (Brief Pain Inventory or an equivalent) - knowledge about cancer-related pain, pain barriers (Barriers Questionnaire) - medication adherence (Medication Adherence Scale, Medication Event Monitoring System or self-report)	Outpatient and inpatient	(Oncology) nurse, research assistant/nurse	stat. sign. differences in favour of education were found for: - pain intensity in 31% of studies - pain interference in 33% of studies *(only evaluated in 40% of included RCTs)* - pain knowledge or barriers in 68% of studies *(only evaluated in 84% of included RCTs)* - medication adherence in 50% of studies *(only evaluated in 23% of included RCTs)*
Prevost et al. 2016 (systematic review of (non-) RCTs	- adults - cancer patients with pain	Patient educational programs (PEP): information, behavioural instructions + advice (by verbal, written, audio- or videotaped, telecare, or computer-aided modalities)	Usual care, general patient education, nutrition education	- pain intensity (NRS) - pain interference (Brief Pain Inventory or an equivalent) - knowledge about cancer-related pain, pain barriers (Barriers Questionnaire) - medication adherence (questionnaires or self-reported)	Ambulatory, home care, and hospital settings	(Oncology) nurse	stat. sign. differences in favour of education were found for: - pain intensity in 52% of studies - pain interference in 12% of studies *(only evaluated in 37% of included RCTs)* - pain knowledge and barriers in 81% of studies *(only evaluated in 70% of included RCTs)* - medication adherence in 45% of studies *(only evaluated in 25% of included RCTs)*
Ling et al. 2012 (review of RCTs)	- adults - cancer-related pain	Educational intervention: information, behavioural instructions and advice by means of verbal, written or audio/video-tape messages	Non-educational treatment, no treatment or usual care	- pain intensity (Brief Pain Inventory, Total Pain Quality Management) - pain interference (Brief Pain Inventory, Total Pain Quality Management)	Outpatient	Healthcare staff	- 50% of studies reported stat. sign. decrease in pain intensity - no stat. sign. results for pain interference
**2. Specific exercise therapy**							
McNeely et al. 2010 (review + meta-analysis of RCTs)	- female adults - breast cancer patients who had surgical removal of breast tumour, axillary lymph node dissection or sentinel node biopsy - during and after cancer treatment	1) Active or active-assisted ROM exercises; 2) Passive ROM/manual stretching exercises; 3) Stretching exercises (including formal exercise interventions such as yoga and Tai Chi Chuan); 4) Strengthening or resistance exercises. Carried out following surgery, during adjuvant treatment and following cancer treatment	1) Early (day 1–3 post-surgery) vs. delayed (day 4 or later post-surgery) 2) usual care/comparison 3) supervised vs. unsupervised	- pain incidence - pain intensity (VAS)	Outpatient and inpatient	Physical therapist, manual therapist, occupational therapist or exercise specialist	*1) Early vs. delayed post-operative exercises:* - no stat. sign. difference in pain incidence at 2w, 1Mo, 6Mo and 2y FU (Bendz et al 2002) and 3Mo FU (Le Vu 1997) 2) *Specific exercises vs. usual care/comparison* - no stat. sign. difference in pain incidence post-intervention (OR: 1.65; 95% CI: 2.50 to 0.81) or at 6Mo FU (OR: 1.51; 95% CI: 2.35 to 0.67) (Beurskens et al 2007) - stat. sign. different decrease in pain intensity: −3.4 vs. −0.5 (*p <* 0.01) at 3Mo; −3.8 vs. −1.0 (*p >* 0.05) at 6Mo (Beurskens et al 2007) 3) *Supervised vs. unsupervised* - no stat. sign. difference in pain intensity post-intervention (MD: −5.40 points; CI: −19.16 to 8.36) (Hwang et al 2008)
De Groef et al. 2015 (review of (pseudo-) RCTs)	- female adults- breast cancer - maximum of 6 weeks postoperative	Active exercises	1) Early (day 1–3 post-surgery) vs. delayed (day 4 or later post-surgery) 2) usual care/comparison/no exercise program	- pain incidence - pain intensity (NRS or VAS)	Outpatient	NS	1) *Early vs. delayed post-operative exercises:* - no stat. sign. differences for pain intensity (reported in only one study, Bendz et al 2002) 2) *Specific exercises vs. usual care* - no stat. sign. difference in pain incidence post-intervention (OR: 1.65; 95% CI: 2.50 to 0.81) or at 6Mo FU (OR: 1.51; 95% CI: 2.35 to 0.67) (Beurskens et al 2007) - stat. sign. different decrease in pain intensity: −3.4 vs. −0.5 (*p <* 0.01) at 3Mo; −3.8 vs. −1.0 (*p >* 0.05) at 6Mo (Beurskens et al 2007)
Carvalho et al. 2012 (review + meta-analyses of RCTs)	- adults - head and neck cancer - during and after cancer treatment - with dysfunction of the shoulder due to having received any type of cancer treatment	1) Active or active-assisted range of motion exercises 2) Passive range of motion exercises 3) Stretching exercises 4) Resistance exercises 5) Proprioceptive neuromuscular facilitation 6) Any other exercise with a focus on shoulder dysfunction treatment or prevention, whether combined or not with pharmacological intervention.	No treatment, usual care, placebo, sham exercises or pharmacological interventions	- pain subscale of the Shoulder Pain and Disability Index (SPADI) (0–100)	Inpatient: Cross Cancer Institute and University of Alberta in Edmonton, Canada (McNeely et al 2004 and 2008)	NS	- stat. sign. beneficial effects for Progressive Strengthening Training (12 weeks) compared to standard care for pain subscale of the SPADI; MD −6.26 95% CI (12.20 to −0.31)
**3. Manual therapy**							
De Groef et al. 2015 (review of (pseudo-) RCTs)	- female adults- breast cancer - max 6 weeks postoperative.	Passive mobilizations	1) Early (day 1–3 post-surgery) vs. delayed (day 4 or later post-surgery) 2) Usual care/comparison/no exercise program	- pain incidence - pain intensity (NRS or VAS)	Outpatient	NS	- pain or sensitivity problems: 74% in no physical therapy vs. 70% mobilisation group vs. 72% massage groups vs. 68% mobilisation and massage group at 3 Mo (*p >* 0.05) - locoregional pain: 5% in mobilization group vs. 13% in no mobilization group (*p =* 0.03) at 8–24 Mo Follow-Up (Le Vu et al., 1997)
Shin et al. 2016 (review + meta-analyses of RCTs)	- adults and children - metastatic, colorectal, advanced, breast, lung, paediatric and non-specified cancer	Massage therapy: tissue manipulation using a carrier oil or blended carrier oil with essential oils (i.e., aromatherapy); excluding touch therapies such as therapeutic touch, acupressure, and reflexology.	No massage	- pain intensity (NRS, VRS or VAS)	Outpatient and inpatient	Trained therapists or not mentioned	- massage significant effect in 1/5 studies on present pain intensity (NRS 0–10): MD −1.60, 95% CI (−2.67 to −0.53)
Boyd et al. 2016 (review + meta-analyses of RCTs)	- adults - metastatic, colorectal, advanced, breast, paediatric and non-specified cancer - with pain	Massage therapy: the systematic manipulation of soft tissue with the hands that positively affects and promotes healing, reduces stress, enhances muscle relaxation, improves local circulation, and creates a sense of well-being.	Sham, no treatment, or active comparator (i.e., participants are actively receiving any type of intervention)	- pain intensity/severity (VAS)	Inpatient, at patient’s or therapist’s home or a hospice	Massage therapist, unspecified therapist, nurse, healing-arts specialist, caregiver, or a researcher trained in massage	- 79% (11/14) of studies showed significant beneficial effects of massage therapy on pain intensity - *meta-analysis massage vs. no treatment including 3 studies*: SMD= −0.20, 95% CI (−0.99 to 0.59); reduction in pain intensity = −5.075, 95% CI (−24.80 to 14.63) - *meta-analysis massage vs. active comparator including 6 studies:* SMD = −0.55, 95% CI (−1.23 to 0.14); reduction in pain intensity = −13.63, 95% CI (−30.78 to 3.5)
**4. General exercise therapy**							
Nakano et al. 2018 (SR and meta-analyses of RCTs	- adults - during and after cancer treatment	1) Aerobic exercise program 2) Resistance exercise program 3) Mixed exercise program	Not receiving any (major) exercise intervention or other interventions (e.g., cognitive behavioural therapy); groups with only attention, relaxation, or education	- EORTC-QLQ-C30 – pain symptom subscale	NS	NS	- overall effect of exercise on EORTC-QLQ-C30 – pain symptom subscale: SMD −0.17, 95% CI (−0.32 to −0.03); p = .02; - no stat. sign. difference among 3 subgroups: 1) aerobic exercise program (4 studies): NS 2) resistance exercise program (3 studies): NS 3) mixed exercise program (4 studies): SMD −0.28; 95% CI (−0.47 to −0.09); p = .005
Mishra et al. 2012 (SR and meta-analyses of RCTs and CCTs)	- adults - after cancer treatment (i.e., survivors) - excluding those who are terminally ill and receiving hospice care	Exercise interventions and any physical activity causing an increase in energy expenditure, and involving a planned or structured movement of the body performed in a systematic manner in terms of frequency, intensity, and duration and is designed to maintain or enhance health-related outcomes	No exercise, another intervention, or usual care (e.g., with no specific exercise program prescribed)	- pain intensity (EORTC-QLQ-C30 – pain symptom subscale or Shoulder Pain and Disability Index (SPADI))	NS	NS	- pain intensity: −0.29 95% CI (−0. 55 to −0.04) standard deviation units after 12 weeks follow-up; (4 studies) *A standard deviation unit is equivalent to about a 28-point change on the QLQ-C30 pain sub-scale*
Mishra et al. 2012 (SR and meta-analyses of RCTs and CCTs)	- adults - during active cancer treatment - excluding those who are terminally ill and receiving hospice care	Exercise interventions and any physical activity causing an increase in energy expenditure, and involving a planned or structured movement of the body performed in a systematic manner in terms of frequency, intensity, and duration and is designed to maintain or enhance health-related outcomes	No exercise, another intervention, or usual care (e.g., with no specific exercise program prescribed)	- Pain intensity (MOS SF-36 – pain subscale, EORTC QLQ-C30 – pain symptom subscale, VAS, MD Anderson Symptom Inventory - pain subscale)	Individual or group, home or facility based	Professionally led or not	- no significant effect was obtained when pooling trials that reported change in pain from baseline to follow-up nor overall pain for follow-up values
**5. Mind-body therapy**							
Pinto-Carral et al. 2018 (SR and meta-analyses of RCTs and CCTs)	- adults - breast cancer - during and after cancer treatment	Pilates exercises: focused on core muscle strengthening, spine flexibility and shoulder girdle range of motion	Other exercise interventions	- Pain intensity (Brief Pain Inventory, VAS)	NS	Specialized pilates centres (outpatient) or at home	- stat. sign effect for pain intensity: SMD −0.48; 95% CI (−0.88 to −0.07)
Danhauer et al 2019 (SR of RCTs)	- adults - breast, prostate, lymphoma colorectal or mixed cancer groups - during and after cancer treatment	Yoga: multicomponent protocols (i.e., movement/postures, breathing and mediation) based on several different yoga types (Anusara, Eischens, Iyengar, Tibetan, Bali, Vivekananda Yoga Anusandhana Samsthana)	Waitlist, usual care or active comparator	- Pain (not further specified)	NS	NS	- 1/1 study stat. sign. improvement of pain *during cancer treatment* - 2/3 studies stat. sign. improvement of pain *after cancer treatment*
Pan et al. 2015 (SR and MA of RCT)	- adults - breast cancer - after active cancer treatment	Tai Chi Chuan (NS)	Psychosocial therapy intervention, standard care, health education	- pain (not specified health-related quality of life questionnaire or SF-36)	NS	NS	- no stat. sign. effect for pain: SMD 0.11; 95% CI (−0.41 to 0.18)

Stat. sign. = Statistically Significant; RCT = Randomized Controlled Trial; SR = Systematic Review; NRS = Numeric Rating Scale; VAS = Visual Analogue Scale; VRS = Verbal Rating Scale; SMD = Standardized Mean Difference; MD = Mean Difference; CI = Confidence Interval; Mo = Months; w = weeks; y = years; EORTC-QLQ-C30 = European Organization for Research and Treatment of Cancer Quality of Life Questionnaire-C30; MOS SF-36 = Medical Outcome Study 36-Item Short From Survey; NS = Not specified.

## References

[1] Torre L.A., Bray F., Siegel R.L., Ferlay J., Lortet-Tieulent J., Jemal A. (2015). Global cancer statistics, 2012. CA Cancer J. Clin..

[2] Ferlay J., Colombet M., Soerjomataram I., Dyba T., Randi G., Bettio M., Gavin A., Visser O., Bray F. (2018). Cancer incidence and mortality patterns in Europe: Estimates for 40 countries and 25 major cancers in 2018. Eur. J. Cancer.

[3] Van den Beuken-van Everdingen M.H., Hochstenbach L.M., Joosten E.A., Tjan-Heijnen V.C., Janssen D.J. (2016). Update on Prevalence of Pain in Patients With Cancer: Systematic Review and Meta-Analysis. J. Pain Symptom Manag..

[4] Pachman D.R., Barton D.L., Swetz K.M., Loprinzi C.L. (2012). Troublesome symptoms in cancer survivors: Fatigue, insomnia, neuropathy, and pain. J. Clin. Oncol..

[5] Glare P.A., Davies P.S., Finlay E., Gulati A., Lemanne D., Moryl N., Oeffinger K.C., Paice J.A., Stubblefield M.D., Syrjala K.L. (2014). Pain in cancer survivors. J. Clin. Oncol..

[6] Bennett M.I., Kaasa S., Barke A., Korwisi B., Rief W., Treede R.D. (2019). The IASP classification of chronic pain for ICD-11: Chronic cancer-related pain. Pain.

[7] Egan M.Y., McEwen S., Sikora L., Chasen M., Fitch M., Eldred S. (2013). Rehabilitation following cancer treatment. Disabil. Rehabil..

[8] Cheville A.L., Mustian K., Winters-Stone K., Zucker D.S., Gamble G.L., Alfano C.M. (2017). Cancer Rehabilitation: An Overview of Current Need, Delivery Models, and Levels of Care. Phys. Med. Rehabil. Clin. N. Am..

[9] Nijs J., Leysen L., Pas R., Adriaenssens N., Meeus M., Hoelen W., Ickmans K., Moloney N. (2016). Treatment of pain following cancer: Applying neuro-immunology in rehabilitation practice. Disabil. Rehabil..

[10] Nijs J., Wijma A.J., Leysen L., Pas R., Willaert W., Hoelen W., Ickmans K., Wilgen C.P.V. (2018). Explaining pain following cancer: A practical guide for clinicians. Braz. J. Phys. Ther..

[11] Koongstvedt P. (2001). The Managed Health Care Handbook.

[12] Louw A., Zimney K., Puentedura E.J., Diener I. (2016). The efficacy of pain neuroscience education on musculoskeletal pain: A systematic review of the literature. Physiother. Theory Pract..

[13] Lee Y.J., Hyun M.K., Jung Y.J., Kang M.J., Keam B., Go S.J. (2014). Effectiveness of education interventions for the management of cancer pain: A systematic review. Asian Pac. J. Cancer Prev..

[14] Bennett M.I., Bagnall A.M., Jose Closs S. (2009). How effective are patient-based educational interventions in the management of cancer pain? Systematic review and meta-analysis. Pain.

[15] Koller A., Miaskowski C., De Geest S., Opitz O., Spichiger E. (2012). A systematic evaluation of content, structure, and efficacy of interventions to improve patients’ self-management of cancer pain. J. Pain Symptom Manag..

[16] Prevost V., Delorme C., Grach M.C., Chvetzoff G., Hureau M. (2016). Therapeutic Education in Improving Cancer Pain Management: A Synthesis of Available Studies. Am. J. Hosp. Palliat. Care.

[17] Oldenmenger W.H., Geerling J.I., Mostovaya I., Vissers K.C.P., de Graeff A., Reyners A.K.L., van der Linden Y.M. (2018). A systematic review of the effectiveness of patient-based educational interventions to improve cancer-related pain. Cancer Treat. Rev..

[18] Ling C.C., Lui L.Y., So W.K. (2012). Do educational interventions improve cancer patients’ quality of life and reduce pain intensity? Quantitative systematic review. J. Adv. Nurs..

[19] McNeely M.L., Campbell K., Ospina M., Rowe B.H., Dabbs K., Klassen T.P., Mackey J., Courneya K. (2010). Exercise interventions for upper-limb dysfunction due to breast cancer treatment. Cochrane Database Syst. Rev..

[20] De Groef A., Van Kampen M., Dieltjens E., Christiaens M.R., Neven P., Geraerts I., Devoogdt N. (2015). Effectiveness of postoperative physical therapy for upper-limb impairments after breast cancer treatment: A systematic review. Arch. Phys. Med. Rehabil..

[21] Carvalho A.P., Vital F.M., Soares B.G. (2012). Exercise interventions for shoulder dysfunction in patients treated for head and neck cancer. Cochrane Database Syst. Rev..

[22] Sierla R., Mun Lee T.S., Black D., Kilbreath S.L. (2013). Lymphedema following breast cancer: Regions affected, severity of symptoms, and benefits of treatment from the patients’ perspective. Clin. J. Oncol. Nurs..

[23] Andersen L.L., Kjaer M., Sogaard K., Hansen L., Kryger A.I., Sjogaard G. (2008). Effect of two contrasting types of physical exercise on chronic neck muscle pain. Arthritis Rheum..

[24] Stubblefield M.D., Keole N. (2013). Upper Body Pain and Functional Disorders in Patients With Breast Cancer. PM & R.

[25] Tatham B., Smith J., Cheifetz O., Gillespie J., Snowden K., Temesy J., Vandenberk L. (2013). The efficacy of exercise therapy in reducing shoulder pain related to breast cancer: A systematic review. Physiother. Can..

[26] Beurskens C.H., van Uden C.J., Strobbe L.J., Oostendorp R.A., Wobbes T. (2007). The efficacy of physiotherapy upon shoulder function following axillary dissection in breast cancer, a randomized controlled study. BMC Cancer.

[27] Hwang J.H., Chang H.J., Shim Y.H., Park W.H., Park W., Huh S.J., Yang J.H. (2008). Effects of supervised exercise therapy in patients receiving radiotherapy for breast cancer. Yonsei Med. J..

[28] Egmond D.L., Mink A.J.F., Vorselaars B.V., Schuitemaker R. (2014). Extermiteiten, Manuele Therapie in Enge en Ruime Zin.

[29] Le Vu B., Dumortier A., Guillaume M.V., Mouriesse H., Barreau-Pouhaer L. (1997). Efficacy of massage and mobilization of the upper limb after surgical treatment of breast cancer. Bull. Du Cancer.

[30] Fellowes D., Barnes K., Wilkinson S. (2004). Aromatherapy and massage for symptom relief in patients with cancer. Cochrane Database Syst. Rev..

[31] Shin E.S., Seo K.H., Lee S.H., Jang J.E., Jung Y.M., Kim M.J., Yeon J.Y. (2016). Massage with or without aromatherapy for symptom relief in people with cancer. Cochrane Database Syst. Rev..

[32] Boyd C., Crawford C., Paat C.F., Price A., Xenakis L., Zhang W., Evidence for Massage Therapy Working Group (2016). The Impact of Massage Therapy on Function in Pain Populations—A Systematic Review and Meta-Analysis of Randomized Controlled Trials: Part II, Cancer Pain Populations. Pain Med..

[33] Mishra S.I., Scherer R.W., Geigle P.M., Berlanstein D.R., Topaloglu O., Gotay C.C., Snyder C. (2012). Exercise interventions on health-related quality of life for cancer survivors. Cochrane Database Syst. Rev..

[34] Mishra S.I., Scherer R.W., Snyder C., Geigle P.M., Berlanstein D.R., Topaloglu O. (2012). Exercise interventions on health-related quality of life for people with cancer during active treatment. Cochrane Database Syst. Rev..

[35] Stout N.L., Baima J., Swisher A.K., Winters-Stone K.M., Welsh J. (2017). A Systematic Review of Exercise Systematic Reviews in the Cancer Literature (2005–2017). PM & R.

[36] Bourke L., Boorjian S.A., Briganti A., Klotz L., Mucci L., Resnick M.J., Rosario D.J., Skolarus T.A., Penson D.F. (2015). Survivorship and improving quality of life in men with prostate cancer. Eur. Urol..

[37] Zeng Y., Huang M., Cheng A.S., Zhou Y., So W.K. (2014). Meta-analysis of the effects of exercise intervention on quality of life in breast cancer survivors. Breast Cancer.

[38] Furmaniak A.C., Menig M., Markes M.H. (2016). Exercise for women receiving adjuvant therapy for breast cancer. Cochrane Database Syst. Rev..

[39] Nakano J., Hashizume K., Fukushima T., Ueno K., Matsuura E., Ikio Y., Ishii S., Morishita S., Tanaka K., Kusuba Y. (2018). Effects of Aerobic and Resistance Exercises on Physical Symptoms in Cancer Patients: A Meta-analysis. Integr. Cancer Ther..

[40] Lahart I.M., Metsios G.S., Nevill A.M., Carmichael A.R. (2018). Physical activity for women with breast cancer after adjuvant therapy. Cochrane Database Syst. Rev..

[41] Lima L.V., Abner T.S., Sluka K.A. (2017). Does exercise increase or decrease pain? Central mechanisms underlying these two phenomena. J. Physiol..

[42] Naugle K.M., Fillingim R.B., Riley J.L. (2012). A meta-analytic review of the hypoalgesic effects of exercise. J. Pain.

[43] Rice D., Nijs J., Kosek E., Wideman T., Hasenbring M.I., Koltyn K., Graven-Nielsen T., Polli A. (2019). Exercise induced hypoalgesia in pain-free and chronic pain populations: State of the art and future directions. J. Pain.

[44] Nahm N., Mee S., Marx G. (2018). Efficacy of management strategies for aromatase inhibitor-induced arthralgia in breast cancer patients: A systematic review. Asian Pac. J. Clin. Oncol..

[45] Yang G.S., Kim H.J., Griffith K.A., Zhu S., Dorsey S.G., Renn C.L. (2017). Interventions for the Treatment of Aromatase Inhibitor-Associated Arthralgia in Breast Cancer Survivors: A Systematic Review and Meta-analysis. Cancer Nurs..

[46] Irwin M.L., Cartmel B., Gross C.P., Ercolano E., Li F., Yao X., Fiellin M., Capozza S., Rothbard M., Zhou Y. (2015). Randomized exercise trial of aromatase inhibitor-induced arthralgia in breast cancer survivors. J. Clin. Oncol..

[47] Husebø A.M.L., Husebø T.L. (2017). Quality of Life and Breast Cancer: How Can Mind–Body Exercise Therapies Help? An Overview Study. Sports.

[48] Pinto-Carral A., Molina A.J., de Pedro A., Ayan C. (2018). Pilates for women with breast cancer: A systematic review and meta-analysis. Complementary Ther. Med..

[49] Danhauer S.C., Addington E.L., Cohen L., Sohl S.J., Van Puymbroeck M., Albinati N.K., Culos-Reed S.N. (2019). Yoga for symptom management in oncology: A review of the evidence base and future directions for research. Cancer.

[50] Pan Y., Yang K., Shi X., Liang H., Zhang F., Lv Q. (2015). Tai chi chuan exercise for patients with breast cancer: A systematic review and meta-analysis. Evid. Based Complementary Altern. Med..

[51] Stout N.L., Binkley J.M., Schmitz K.H., Andrews K., Hayes S.C., Campbell K.L., McNeely M.L., Soballe P.W., Berger A.M., Cheville A.L. (2012). A prospective surveillance model for rehabilitation for women with breast cancer. Cancer.

[52] Holland J.C., Reznik I. (2005). Pathways for psychosocial care of cancer survivors. Cancer.

[53] Coccia P.F., Pappo A.S., Beaupin L., Borges V.F., Borinstein S.C., Chugh R., Dinner S., Folbrecht J., Frazier A.L., Goldsby R. (2018). Adolescent and Young Adult Oncology, Version 2.2018, NCCN Clinical Practice Guidelines in Oncology. J. Natl. Compr. Cancer Netw..

[54] Kvale E., Urba S.G. (2014). NCCN guidelines for survivorship expanded to address two common conditions. J. Natl. Compr. Cancer Netw..

[55] Alfano C.M., Kent E.E., Padgett L.S., Grimes M., de Moor J.S. (2017). Making Cancer Rehabilitation Services Work for Cancer Patients: Recommendations for Research and Practice to Improve Employment Outcomes. PM & R.

[56] Smart K.M., Blake C., Staines A., Doody C. (2011). The Discriminative validity of “nociceptive,” “peripheral neuropathic,” and “central sensitization” as mechanisms-based classifications of musculoskeletal pain. Clin. J. Pain.

[57] Chimenti R.L., Frey-Law L.A., Sluka K.A. (2018). A Mechanism-Based Approach to Physical Therapist Management of Pain. Phys. Ther..

[58] Nijs J., Leysen L., Adriaenssens N., Aguilar Ferrandiz M.E., Devoogdt N., Tassenoy A., Ickmans K., Goubert D., van Wilgen C.P., Wijma A.J. (2016). Pain following cancer treatment: Guidelines for the clinical classification of predominant neuropathic, nociceptive and central sensitization pain. Acta Oncol..

[59] Fernandez-Lao C., Cantarero-Villanueva I., Fernandez-de-las-Penas C., Del-Moral-Avila R., Menjon-Beltran S., Arroyo-Morales M. (2011). Widespread mechanical pain hypersensitivity as a sign of central sensitization after breast cancer surgery: Comparison between mastectomy and lumpectomy. Pain Med..

[60] Andrykowski M.A., Curran S.L., Carpenter J.S., Studts J.L., Cunningham L., McGrath P.C., Sloan D.A., Kenady D.E. (1999). Rheumatoid symptoms following breast cancer treatment: A controlled comparison. J. Pain Symptom Manag..

[61] Kosek E., Cohen M., Baron R., Gebhart G.F., Mico J.A., Rice A.S., Rief W., Sluka A.K. (2016). Do we need a third mechanistic descriptor for chronic pain states?. Pain.

[62] Niravath P. (2013). Aromatase inhibitor-induced arthralgia: A review. Ann. Oncol..

[63] Edwards R.R., Dworkin R.H., Sullivan M.D., Turk D.C., Wasan A.D. (2016). The Role of Psychosocial Processes in the Development and Maintenance of Chronic Pain. J. Pain.

[64] Heathcote L.C., Eccleston C. (2017). Pain and cancer survival: A cognitive-affective model of symptom appraisal and the uncertain threat of disease recurrence. Pain.

[65] Jones L.W., Eves N.D., Haykowsky M., Freedland S.J., Mackey J.R. (2009). Exercise intolerance in cancer and the role of exercise therapy to reverse dysfunction. Lancet Oncol..

[66] Ciuca A., Baban A. (2017). Psychological factors and psychosocial interventions for cancer related pain. Rom. J. Intern. Med..

[67] Kiasuwa Mbengi R., Otter R., Mortelmans K., Arbyn M., Van Oyen H., Bouland C., de Brouwer C. (2016). Barriers and opportunities for return-to-work of cancer survivors: Time for action—rapid review and expert consultation. Syst. Rev..

[68] Rock C.L., Doyle C., Demark-Wahnefried W., Meyerhardt J., Courneya K.S., Schwartz A.L., Bandera E.V., Hamilton K.K., Grant B., McCullough M. (2012). Nutrition and physical activity guidelines for cancer survivors. CA Cancer J. Clin..

[69] Davis S.W., Oakley-Girvan I. (2017). Achieving value in mobile health applications for cancer survivors. J. Cancer Surviv..

[70] Hernandez Silva E., Lawler S., Langbecker D. (2019). The effectiveness of mHealth for self-management in improving pain, psychological distress, fatigue, and sleep in cancer survivors: A systematic review. J. Cancer Surviv..

[71] Harder H., Holroyd P., Burkinshaw L., Watten P., Zammit C., Harris P.R., Good A., Jenkins V. (2017). A user-centred approach to developing bWell, a mobile app for arm and shoulder exercises after breast cancer treatment. J. Cancer Surviv..

[72] Kuijpers W., Groen W.G., Aaronson N.K., van Harten W.H. (2013). A systematic review of web-based interventions for patient empowerment and physical activity in chronic diseases: Relevance for cancer survivors. J. Med. Internet Res..

[73] Leysen L., Adriaenssens N., Nijs J., Pas R., Bilterys T., Vermeir S., Lahousse A., Beckwee D. (2018). Chronic pain in breast cancer survivors: Nociceptive, neuropathic or central sensitization pain?. Pain Pract..

[74] Kumar S.P., Prasad K., Kumar V.K., Shenoy K., Sisodia V. (2013). Mechanism-based Classification and Physical Therapy Management of Persons with Cancer Pain: A Prospective Case Series. Indian J. Palliat. Care.

[75] Dworkin R.H., Turk D.C., Farrar J.T., Haythornthwaite J.A., Jensen M.P., Katz N.P., Kerns R.D., Stucki G., Allen R.R., Bellamy N. (2005). Core outcome measures for chronic pain clinical trials: IMMPACT recommendations. Pain.

[76] Sun Y., Shigaki C.L., Armer J.M. (2017). Return to work among breast cancer survivors: A literature review. Support. Care Cancer.

[77] Mehnert A. (2011). Employment and work-related issues in cancer survivors. Crit. Rev. Oncol. Hematol..

[78] Dunn J., Yeo E., Moghaddampour P., Chau B., Humbert S. (2017). Virtual and augmented reality in the treatment of phantom limb pain: A literature review. NeuroRehabilitation.

[79] Maggio M.G., Russo M., Cuzzola M.F., Destro M., La Rosa G., Molonia F., Bramanti P., Lombardo G., De Luca R., Calabro R.S. (2019). Virtual reality in multiple sclerosis rehabilitation: A review on cognitive and motor outcomes. J. Clin. Neurosci..

[80] Pozeg P., Palluel E., Ronchi R., Solca M., Al-Khodairy A.W., Jordan X., Kassouha A., Blanke O. (2017). Virtual reality improves embodiment and neuropathic pain caused by spinal cord injury. Neurology.

[81] Arane K., Behboudi A., Goldman R.D. (2017). Virtual reality for pain and anxiety management in children. Can. Fam. Phys..

